# Trans-ethnic predicted expression genome-wide association analysis identifies a gene for estrogen receptor-negative breast cancer

**DOI:** 10.1371/journal.pgen.1006727

**Published:** 2017-09-28

**Authors:** Guimin Gao, Brandon L. Pierce, Olufunmilayo I. Olopade, Hae Kyung Im, Dezheng Huo

**Affiliations:** 1 Department of Public Health Sciences, University of Chicago, Chicago, United States of America; 2 Department of Human Genetics, University of Chicago, Chicago, United States of America; 3 Section of Hematology and Oncology, Department of Medicine, University of Chicago, Chicago, United States of America; 4 Section of Genetic Medicine, Department of Medicine, University of Chicago, Chicago, United States of America; Case Western Reserve University School of Medicine, UNITED STATES

## Abstract

Genome-wide association studies (GWAS) have identified more than 90 susceptibility loci for breast cancer, but the underlying biology of those associations needs to be further elucidated. More genetic factors for breast cancer are yet to be identified but sample size constraints preclude the identification of individual genetic variants with weak effects using traditional GWAS methods. To address this challenge, we utilized a gene-level expression-based method, implemented in the MetaXcan software, to predict gene expression levels for 11,536 genes using expression quantitative trait loci and examine the genetically-predicted expression of specific genes for association with overall breast cancer risk and estrogen receptor (ER)-negative breast cancer risk. Using GWAS datasets from a Challenge launched by National Cancer Institute, we identified *TP53INP2* (tumor protein p53-inducible nuclear protein 2) at 20q11.22 to be significantly associated with ER-negative breast cancer (Z = -5.013, p = 5.35×10^−7^, Bonferroni threshold = 4.33×10^−6^). The association was consistent across four GWAS datasets, representing European, African and Asian ancestry populations. There are 6 single nucleotide polymorphisms (SNPs) included in the prediction of *TP53INP2* expression and five of them were associated with estrogen-receptor negative breast cancer, although none of the SNP-level associations reached genome-wide significance. We conducted a replication study using a dataset outside of the Challenge, and found the association between *TP53INP2* and ER-negative breast cancer was significant (p = 5.07x10^-3^). Expression of *HP* (16q22.2) showed a suggestive association with ER-negative breast cancer in the discovery phase (Z = 4.30, p = 1.70x10^-5^) although the association was not significant after Bonferroni adjustment. Of the 249 genes that are 250 kb within known breast cancer susceptibility loci identified from previous GWAS, 20 genes (8.0%) were statistically significant associated with ER-negative breast cancer (p<0.05), compared to 582 (5.2%) of 11,287 genes that are not close to previous GWAS loci. This study demonstrated that expression-based gene mapping is a promising approach for identifying cancer susceptibility genes.

## Author summary

Although individual genetic variant-based genome-wide association studies have greatly increased our understanding of the genetic susceptibility to breast cancer, the genetic variants identified to date account for a relatively small proportion of the heritability. Shifting the focus of analysis from individual genetic variants to genes or gene sets could lead to the identification of novel genes involved in breast cancer risk. Here, we take advantage of a recently developed gene-level expression-based association method MetaXcan to examine the association of genetically-predicted expression levels for 11,536 genes across the human genome with breast cancer risk. The MetaXcan method uses external information on the effects of genetic variants on gene expression. We show that the *TP53INP2* gene on human chromosome 20 is significantly associated with estrogen-receptor negative breast cancer (P = 5.35×10^−7^, Bonferroni threshold = 4.33×10^−6^). The association is consistent across analyses of four datasets, representing European, African and Asian ancestry populations. As a downstream gene of p53, *TP53INP2* may affect breast cancer risk through p53 signaling pathway. Furthermore, *TP53INP2*, also known as *DOR* (Diabetes And Obesity-Regulated Gene), has been linked to obesity and diabetes, suggesting a novel biological pathway for the known association between obesity and breast cancer risk.

## Introduction

Breast cancer is the most common cancer in women in the United States and in the world [1]. It is a heterogeneous disease and the two main subgroups of breast cancer are estrogen receptor (ER)-positive and ER-negative cancer. Genome-wide association studies (GWAS) have identified more than 90 susceptibility loci for breast cancer [2–20], with only a few loci specific for ER-negative breast cancer [3,15,17]. Susceptibility loci for ER-positive loci are often the same as loci for overall breast cancer risk because most of breast cancers are ER-positive, especially in women of European or Asian ancestry [2,4,19].

Women of African ancestry are more likely to be diagnosed with ER-negative breast cancer compared to women of non-African ancestry [21–23]. To date, breast cancer GWAS have been conducted primarily in populations of European ancestry. The difference in linkage disequilibrium (LD) patterns and allele frequencies across ancestry groups may explain the apparent inconsistencies in GWAS findings from studies of women of European ancestry as compared to studies of women of African ancestry [24,25]. The strength and the direction of the association between causal variants and disease are expected to be consistent across populations, and thus cross-population validation provides further evidence of causation. In addition, trans-ancestry analysis could identify novel breast cancer susceptibility variants [26].

The variants discovered by previous GWAS along with previously known high-penetrance genes explain only a modest proportion of the heritability of breast cancer [2]. More genetic factors for breast cancer are yet to be identified, but power for discovery of new loci is limited by the sample size of existing GWASs. Moreover, the biologic significance of the variants identified by GWAS and the genes on which they act, are often unknown. Single nucleoid polymorphisms (SNPs) associated with disease traits are more likely to be expression quantitative trait loci (eQTLs) [27], and regulatory variants can explain a large proportion of disease heritability [28]. Therefore, genes regulated by eQTLs can be used as an enrichment analysis unit to identify more genetic risk factors for breast cancer. Recently, gene-based approaches using eQTL information, such as PrediXcan, have been proposed, which can reduce the multiple testing burden in genome-wide analyses and have been used to identify novel genes for autoimmune diseases [29]. PrediXcan uses individual-level data to estimate the correlation between genetically predicted levels of gene expression and human traits to prioritize causal genes. MetaXcan computes the same correlation as PrediXcan, but does so using summary statistics from GWAS, which are much more readily accessible than individual level data [30].

To identify novel genes involved in breast cancer susceptibility, we utilized a gene-level expression-based association method, implemented in the MetaXcan software [30], to infer gene expression levels using summary statistics from five GWASs. We used an additive prediction model of gene-expression levels trained in Depression Genes and Network (DGN) data [31] and examined the predicted expression of specific genes for association with overall breast cancer risk and estrogen receptor-negative breast cancer risk. The GWAS datasets were made available in dbGaP (https://www.ncbi.nlm.nih.gov/gap) through “Up For A Challenge (U4C)–Stimulating Innovation in Breast Cancer Genetic Epidemiology” launched by the National Cancer Institute. The DGN data included RNA sequencing data from whole blood of 922 genotyped individuals (463 cases of major depressive disorder and 459 controls), all of European ancestry. These individuals consisted of 274 males and 648 females with ages ranged from 21 to 60.

## Results

**Fig 1:**
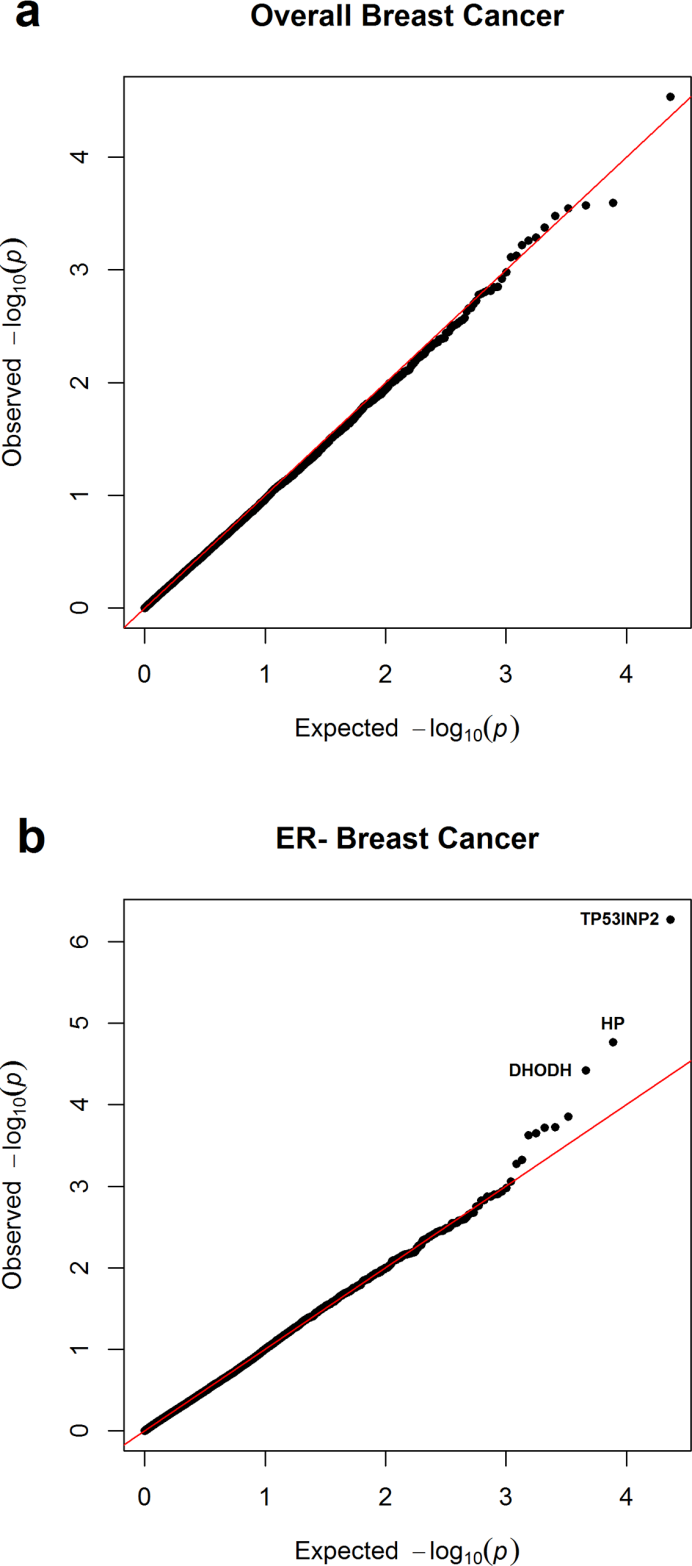
**Quantile-quantile plot of gene-based association P values for (a) overall and (b) estrogen receptor negative breast cancer.** Red line shows the null distribution of P values.

Using logistic regression, we first conducted SNP-level GWAS analysis for overall breast cancer risk among 8605 breast cancer cases and 8095 controls, and for ER-negative breast cancer risk among 3879 cases and 10213 controls. The analyses were performed for each of the five GWAS datasets separately and summary statistics including log odds ratios and standard errors were generated. These summary statistics for each dataset were input to the software MetaXcan [30] to perform genome-wide gene-level expression association tests for 11,536 genes. Then, we performed meta-analysis of the results from individual MetaXcan analyses. Quantile-quantile plots of P-values from the meta-analysis showed little inflation (**Fig 1**). For overall breast cancer risk, there was no gene with a P-value that deviated from the null distribution (**Fig 1A**), but for ER-negative breast cancer risk analysis, there were several genes with P-values smaller than expected, including *TP53INP2*, *HP*, and *DHODH* (**Fig 1B**).

**Table 1** lists the top genes with P-values less than 10^−3^ in the analyses of association between predicted gene expressions and overall breast cancer risk. The sign of Z score indicates the direction of association between genetically-predicted expression and breast cancer risk. None of the genes reached genome-wide significance when a Bonferroni threshold (*α* = 4.33x10^-6^) was used.

**Table 1:** Top genes with P-values < 10^−3^ in analyses of association between predicted gene expressions and overall breast cancer risk*.

Gene	Cytoband	SNPs in predictor	AABC		CGEMS		ROOT		SBCGS		Total		
			Z score	P value	Z score	P value	Z score	P value	Z score	P value	Z score	P value	FDR
**TP53INP2**	**20q11.22**	6	**-2.536**	**1.12E-02**	**-0.953**	**3.41E-01**	**-3.023**	**2.50E-03**	**-1.683**	**9.23E-02**	**-4.180**	**2.91E-05**	0.34
BAG3	10q26.11	18	-2.109	3.49E-02	-1.145	2.52E-01	-3.003	2.67E-03	-1.074	2.83E-01	-3.660	2.52E-04	0.77
POLN	4p16.3	39	-2.291	2.20E-02	-2.614	8.96E-03	-0.942	3.46E-01	-1.624	1.04E-01	-3.644	2.68E-04	0.77
WDR37	10p15.3	9	-1.637	1.02E-01	-0.747	4.55E-01	-0.292	7.70E-01	-4.144	3.42E-05	-3.629	2.84E-04	0.77
TTLL5	14q24.3	26	2.717	6.59E-03	2.087	3.69E-02	1.189	2.35E-01	1.206	2.28E-01	3.588	3.33E-04	0.77
HP	16q22.2	19	2.424	1.53E-02	1.961	4.99E-02	1.598	1.10E-01	1.147	2.52E-01	3.529	4.18E-04	0.77
VTI1B	14q24.1	1	1.433	1.52E-01	2.790	5.28E-03	0.902	3.67E-01	2.151	3.15E-02	3.471	5.18E-04	0.77
HLA-DMA	6p21.32	30	-2.001	4.54E-02	-0.756	4.50E-01	-1.603	1.09E-01	-2.293	2.19E-02	-3.456	5.47E-04	0.77
MYOM2	8p23.3	109	2.338	1.94E-02	-0.051	9.59E-01	2.153	3.14E-02	1.955	5.06E-02	3.430	6.04E-04	0.77
MYO9B	19p13.11	6	1.643	1.00E-01	0.887	3.75E-01	1.473	1.41E-01	2.549	1.08E-02	3.373	7.44E-04	0.81
ZNF202	11q24.1	15	2.214	2.69E-02	1.675	9.39E-02	0.003	9.98E-01	2.644	8.20E-03	3.363	7.71E-04	0.81

*Bonferroni threshold = 4.33×10^−6^.

FDR, false discovery rate.

Of the 249 genes that are 250 kb within known susceptibility loci identified from previous breast cancer GWAS [2–4,17,32], 12 genes (4.8%) were statistically significant associated with overall breast cancer risk at nominal significance level of 0.05, compared to 497 (4.4%) of 11,287 genes that are not close to previous GWAS loci (P for enrichment = 0.75).

**Table 2** lists the genes with P-values less than 10^−3^ in the ER-negative breast cancer analysis. *TP53INP2* was the top gene (P = 5.35x10^-7^), which surpassed the Bonferroni-corrected p-value threshold (*α* = 4.33x10^-6^). The false discovery rate for *TP53INP2* was 0.0062. Higher genetically-predicted *TP53INP2* expression was associated with lower risk of ER-negative breast cancer. The gene with the second smallest P-value was *HP*, which had p-value of 1.70x10^-5^, close to but not significant after Bonferroni correction. The false discovery rate for the *HP* gene was 0.098. For the *HP* gene, higher expression was associated with higher risk of ER-negative breast cancer. Both genes are novel and no previous studies have found association between these two genes and breast cancer risk.

**Table 2:** Top genes with P-values < 10^−3^ in analyses of association between predicted gene expressions and ER-negative breast cancer risk*.

Gene	Cytoband	SNPs in predictor	AABC		BPC3		ROOT		SBCGS		Total		
			Z score	P value	Z score	P value	Z score	P value	Z score	P value	Z score	P value	FDR
**TP53INP2**	**20q11.22**	**6**	**-3.708**	**2.09E-04**	**-2.919**	**3.51E-03**	**-2.703**	**6.87E-03**	**-0.417**	**6.77E-01**	**-5.013**	**5.35E-07**	0.0062
**HP**	**16q22.2**	**20**	**1.424**	**1.54E-01**	**3.302**	**9.61E-04**	**1.851**	**6.41E-02**	**1.749**	**8.03E-02**	**4.300**	**1.70E-05**	0.098
DHODH	16q22.2	58	-1.121	2.62E-01	-4.700	2.61E-06	1.020	3.08E-01	-1.859	6.31E-02	-4.119	3.80E-05	0.15
YJEFN3	19p13.11	20	-2.650	8.05E-03	-2.797	5.16E-03	0.154	8.78E-01	-1.549	1.21E-01	-3.810	1.39E-04	0.34
MAP1LC3A	20q11.22	49	-2.077	3.78E-02	-2.922	3.48E-03	-1.751	7.99E-02	-0.157	8.75E-01	-3.734	1.88E-04	0.34
DPY19L1	7p14.2	24	2.188	2.87E-02	3.035	2.41E-03	0.672	5.01E-01	0.791	4.29E-01	3.731	1.91E-04	0.34
GCOM1	15q21.3	75	-1.841	6.56E-02	-3.295	9.85E-04	-0.854	3.93E-01	-0.525	5.99E-01	-3.689	2.25E-04	0.34
AMOTL1	11q21	14	2.155	3.12E-02	2.118	3.42E-02	0.448	6.54E-01	2.509	1.21E-02	3.675	2.38E-04	0.34
ITCH	20q11.22	12	-1.597	1.10E-01	-3.861	1.13E-04	0.203	8.39E-01	-0.318	7.51E-01	-3.494	4.77E-04	0.61
TRPC4AP	20q11.22	26	2.385	1.71E-02	1.899	5.76E-02	2.536	1.12E-02	0.127	8.99E-01	3.466	5.28E-04	0.61
SNX24	5q23.2	3	-0.902	3.67E-01	-2.235	2.54E-02	-1.612	1.07E-01	-2.022	4.32E-02	-3.327	8.77E-04	0.91

*Bonferroni threshold = 4.33×10^−6^

FDR, false discovery rate

Of the 249 genes that are 250 kb within known breast cancer susceptibility loci identified from previous GWAS, 20 genes (8.0%) were statistically significant associated with ER-negative breast cancer (p<0.05), compared to 582 (5.2%) of 11,287 genes that are not close to previous GWAS loci (P for enrichment = 0.044), suggesting a moderate enrichment for genes close to known susceptibility loci.

There were six SNPs included in the prediction of the expression of the *TP53INP2* gene, from 367 kb upstream to 159 kb downstream of the gene (**Table 3**). Five of the six SNPs (except for rs8116198) were associated with overall breast cancer risk and ER-negative breast cancer risk (at the nominal level of α = 0.05), and the effects were consistently across studies (none of the heterogeneity tests were significant). These associations were more significant for ER-negative breast cancer risk (p values ranging from 5.0x10^-4^ to 1.8x10^-6^) than for overall breast cancer risk (7.0x10^-4^ to 1.4x10^-4^). None of the SNP-level associations reached traditional genome-wide significance, thus they have not been reported in previous GWAS publications. However, our study showed the aggregate effects of these SNPs were significantly associated with ER-negative breast cancer after Bonferroni correction. We noticed that one of the six SNPs, rs8116198, is monomorphic in the SBCGS data. Therefore, when MetaXcan was applied to the SBCGS data, the prediction of *TP53INP2* expression was based on only five SNPs. To make our results more robust to missing and low quality genotypes, in the DGN prediction model, we used elastic net with 0.5 as the mixing parameter, which sets the degree of mixing between ridge regression and LASSO. In addition, the SNPs in the prediction were not necessarily causal but could be in LD with the causal SNPs.

**Table 3:** *TP53INP2*-related SNPs and their association with breast cancer risk.

	Pos. at chr20 /from TP53INP2*			Overall		ER-negative	
SNP		Test/ref allele	Study	OR (95% CI)	P	OR (95% CI)	P
rs1205339	32,924,967	G/A	BPC3			1.17 (1.05–1.31)	5.9E-03
	-367,127		CGEMS	1.05 (0.91–1.23)	0.49		
			AABC	1.14 (1.03–1.27)	0.013	1.29 (1.12–1.50)	5.6E-04
			ROOT	1.19 (1.04–1.36)	0.012	1.39 (1.10–1.76)	5.6E-03
			SBCGS	1.07 (0.97–1.18)	0.18	1.06 (0.89–1.26)	0.51
			meta	1.11 (1.05–1.18)	4.2E-04	1.20 (1.11–1.29)	1.8E-06
rs4911154	32,996,101	A/G	BPC3			1.16 (1.04–1.30)	0.01
	-295,993		CGEMS	1.04 (0.89–1.21)	0.65		
			AABC	1.15 (1.03–1.28)	0.014	1.32 (1.14–1.54)	2.9E-04
			ROOT	1.23 (1.07–1.42)	3.5E-03	1.39 (1.09–1.78)	8.3E-03
			SBCGS	1.09 (0.98–1.21)	0.11	1.06 (0.88–1.28)	0.54
			meta	1.13 (1.06–1.20)	1.6E-04	1.20 (1.11–1.30)	2.6E-06
rs8116198†	33,114,201	G/A	BPC3			0.92 (0.80–1.05)	0.21
	-177,893		CGEMS	0.94 (0.78–1.13)	0.5		
			AABC	1.04 (0.82–1.33)	0.73	1.11 (0.79–1.57)	0.54
			ROOT	0.67 (0.45–1.00)	0.052	0.87 (0.49–1.54)	0.63
			meta	0.93 (0.81–1.07)	0.33	0.92 (0.80–1.04)	0.30
rs6058107	33,288,546	C/T	BPC3			0.87 (0.78–0.97)	8.7E-03
	-3,548		CGEMS	0.92 (0.80–1.06)	0.27		
			AABC	0.91 (0.83–1.01)	0.072	0.84 (0.73–0.96)	0.014
			ROOT	0.83 (0.73–0.94)	4.00E-03	0.80 (0.64–0.99)	0.041
			SBCGS	0.92 (0.84–1.00)	0.057	1.03 (0.88–1.20)	0.71
			meta	0.90 (0.85–0.95)	1.4E-04	0.90 (0.83–0.97)	5.0E-04
rs6060047	33,367,400	G/T	BPC3			0.87 (0.77–0.97)	0.017
	75,306		CGEMS	0.91 (0.78–1.07)	0.27		.
			AABC	0.88 (0.79–0.98)	0.016	0.75 (0.65–0.86)	7.5E-05
			ROOT	0.83 (0.73–0.95)	6.10E-03	0.76 (0.60–0.96)	0.019
			SBCGS	0.94 (0.86–1.03)	0.21	0.98 (0.83–1.16)	0.81
			meta	0.90 (0.85–0.95)	2.1E-04	0.84 (0.78–0.91)	7.3E-06
rs11546155	33,451,148	A/G	BPC3			1.19 (1.04–1.35)	9.1E-03
	159,054		CGEMS	1.12 (0.94–1.34)	0.21		
			AABC	1.14 (1.02–1.27)	0.023	1.32 (1.14–1.54)	3.3E-04
			ROOT	1.11 (0.96–1.28)	0.15	1.18 (0.93–1.51)	0.18
			SBCGS	1.16 (0.98–1.38)	0.089	1.26 (0.94–1.70)	0.13
			meta	1.13 (1.05–1.21)	7.0E-04	1.23 (1.13–1.35)	2.0E-06

* NCBI 37 and from transcription starting site of *TP53INP2*

† rs8116198 is monomorphic in Asian population.

None of the tests for heterogeneity across studies was significant.

OR, odds ratio; CI, confidence intervals; ER, estrogen receptor

**Fig 2:**
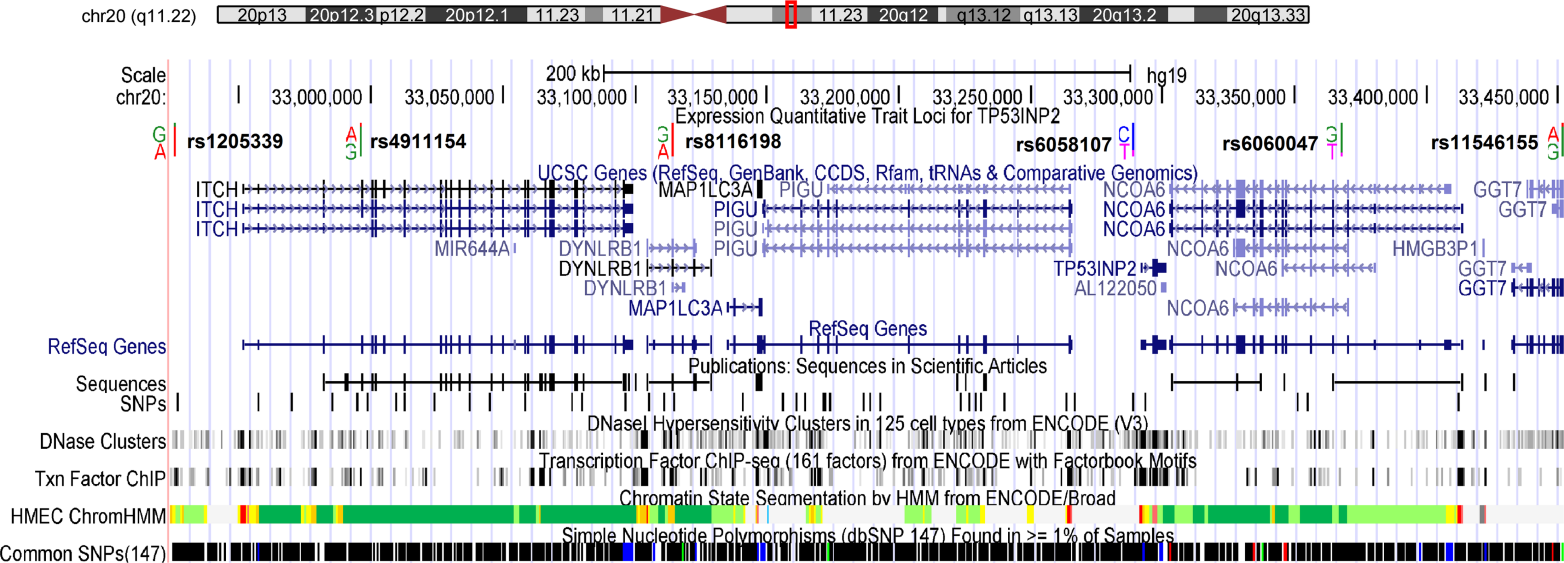
The 20q11.22 locus spanned for expression quantitative trait loci of *TN53INT2*, and analysis of regulation enhancer with data from ENCODE through UCSC Genome Browser, including transcription factor binding sites and human mammary epithelial cells (HMEC) histone modification marks. Chromosomal coordinates are in NCBI build 37.

**Fig 2** shows positions of the 6 eQTL SNPs for *TP53INP2* in the cytoband 20q11.22. Interestingly, there are several other genes in this region that were associated with ER-negative breast cancer, including *MAP1LC3A*, *ITCH*, and *TRPC4AP* (**Fig 2** and **Table 2**). The 6 SNPs are located either in enhancer elements or in promotor regions (**Table 4**). The promotor/enhancer features of 4 SNPs were found in human mammary epithelial cells (HMEC) and breast variant human mammary epithelial cells (HMEC.35), and the enrichment was statistically significant for both cell types (both p<0.03).

**Table 4:** Regulatory element annotation of variants that predicted expression of *TP53INP2* using HaploReg [33].

Variant	Position*	Promoter histone marks†	Enhancer histone marks†	DNAse hypersensitivity	Proteins bound	Motifs changed by the variant
rs1205339	-367,127		6 tissues including breast and blood (HMEC, MYO, HMEC.35)			ATF2, Mef2, Pax-4, Pou1f1, TATA
rs4911154	-295,993		Liver	Blood, liver	TCF4	RFX5
rs8116198	-177,893		24 tissues including breast and blood (HMEC, MYO, HMEC.35)		POL2, TBP, TR4	Rad21
rs6058107	-3,548	24 tissues including breast and blood (HMEC, MYO, HMEC.35)‡		28 tissues including breast and blood‡		AP-1,NF-E2
rs6060047	75,306		Multiple tissue types including blood and breast (HMEC, MYO, HMEC.35)‡	Multiple tissue types including blood		BATF, GCNF, HNF1, Irf, STAT
rs11546155	159,054	2 tissue types	4 tissue types			NRSF, Pou5f1, RXRA, Sin3Ak-20

* base pair from the transcription starting site of *TP53INP2*

† Normal mammary or breast cancer cell lines are indicated in parenthesis. HMEC.35: breast variant human mammary epithelial cells; MYO: breast myoepithelial primary cells; HMEC: mammary epithelial primary cells (vMHEC)

‡ Variants in strong linkage disequilibrium

There were 20 SNPs included in the prediction of the expression of the *HP* gene (**S1 Table**). Thirteen of the 20 SNPs were associated with overall breast cancer risk and 17 were associated with the risk of ER-negative breast cancer (at the nominal level of α = 0.05), quite consistently across populations (none of the heterogeneity tests were significant). The strengths of their associations were stronger for ER-negative breast cancer risk than for overall breast cancer risk. Interestingly, none of the associations for individual SNPs reached genome-wide significance, thus they have not been reported in previous GWAS publications.

We used summary results from GAME-ON GWAS (http://gameon.dfci.harvard.edu) to replicate our study findings from the U4C. All the six eQTLs for the *TP53INP2* gene were available in GAME-ON (**Table 5**). Five of the six SNPs that were associated with ER-negative breast cancer in the discovery phase (using U4C datasets) were all statistically significant in GAME-ON at the nominal 0.05 significance level. Gene-level test of *TP53INP2* from MetaXcan gave a Z-score of -2.803 (p = 5.1×10^−3^) for ER-negative breast cancer in GAME-ON. The gene-level test for overall breast cancer risk was not significant in GAME-ON (Z-score = -1.627, p = 0.10). Because the GAME-ON ER-negative data included the BPC3 dataset, in order to show the independent replication, we tested association in the U4C ER-negative data excluding BPC3, and found the Z-score for the *TP53INP2* gene was -4.127 (p = 3.67×10^−5^).

**Table 5:** GAME-ON replication for SNPs related to the *TP53INP2* gene.

	Test/ref allele	Study phase	Overall		ER-negative*	
SNP			OR (95% CI)	P	OR (95% CI)	P
rs11546155	A/G	U4C	1.13 (1.05–1.21)	7.0E-04	1.28 (1.14–1.44)	5.0E-05
		GAME-ON	1.05 (0.99–1.10)	0.11	1.13 (1.03–1.25)	0.013
rs1205339	G/A	U4C	1.11 (1.05–1.18)	4.1E-04	1.22 (1.11–1.35)	8.0E-05
		GAME-ON	1.02 (0.98–1.07)	0.3	1.09 (1.01–1.17)	0.021
rs4911154	A/G	U4C	1.13 (1.06–1.20)	1.6E-04	1.25 (1.12–1.39)	5.5E-05
		GAME-ON	1.02 (0.98–1.07)	0.31	1.09 (1.01–1.17)	0.02
rs6058107	C/T	U4C	0.90 (0.85–0.95)	1.5E-04	0.90 (0.82–0.98)	0.021
		GAME-ON	0.96 (0.92–1.00)	0.045	0.91 (0.85–0.97)	5.0E-03
rs6060047	G/T	U4C	0.90 (0.85–0.95)	2.1E-04	0.82 (0.75–0.91)	1.2E-04
		GAME-ON	0.96 (0.91–1.00)	0.066	0.90 (0.84–0.97)	7.7E-03
rs8116198	G/A	U4C	0.93 (0.81–1.07)	0.33	1.04 (0.78–1.40)	0.79
		GAME-ON	0.97 (0.91–1.03)	0.28	0.94 (0.84–1.05)	0.28

*The overlapping study (BPC3) was removed from the meta-analysis in the discovery phase (U4C).

OR, odds ratio; CI, confidence interval; ER, estrogen receptor

For the *HP* gene, the direction of association for 19 SNPs (out of 20) were consistent between U4C and GAME-ON for ER-negative breast cancer risk, but only 2 SNPs were statistically significant at nominal 0.05 level in GAME-ON (**S2 Table**). None of the SNPs were significantly associated with overall breast cancer risk in GAME-ON. In the gene-based analysis using GAME-ON data, the Z-score for overall breast cancer risk was 1.769 (p = 0.077) and the Z-score for ER-negative breast cancer risk was 2.02 (p = 0.043). In addition, we tested this association in the U4C ER-negative data excluding BPC3, and found the Z-score for the *HP* gene was 2.81 (p = 5.1×10^−3^).

## Discussion

In this gene-level expression-based genome-wide association analysis of five breast cancer GWAS datasets composed of individuals of diverse ancestry, we identified *TP53INP2* (20q11.22) as gene with genetically-determined expression that is associated with ER-negative breast cancer. The gene-based analysis of aggregated eQTLs for a particular gene as an analysis unit can reduce the burden of multiple testing and provide a direction of association between expression of a specific gene and disease risk. We found that increased expression of *TP53INP2* expression in whole blood was associated with a decrease in ER-negative breast cancer risk. In addition, we identified the *HP* gene in the 16q22.2 regions to have expression levels that are positively associated with ER- negative breast cancer.

The *TP53INP2* gene (tumor protein p53-inducible nuclear protein 2) is 9150 base pairs long and codes for a 220 amino acid protein, which is a dual regulator of transcription and autophagy and is required for autophagosome formation and processing. One experimental study showed that overexpression of *TP53INP2* severely attenuated proliferative and invasive capacity of melanoma cells, via p53 signaling and lysosomal pathways [34]. This inverse correlation between *TP53INP2* expression and cancer proliferation is consistent with our finding that *TP53INP2* expression inversely correlated with breast cancer risk. P53 is a transcription factor for *TP53INP2*, and *TP53* plays an important role in development of multiple cancers. Germline *TP53* mutations cause Li-Fraumeni syndrome, characterized as a cluster of cancers including breast cancer [35]. Somatic TP53 mutation is a common event in ER-negative breast cancer [36]. As a downstream gene of p53, *TP53INP2* may affect breast cancer risk through p53 signaling pathway. Also, known as *DOR* (diabetes- and obesity-regulated gene), *TP53INP2* has been linked to obesity and diabetes [37]. *TP53INP2* is also associated with triglycerides and cholesterol level. One experimental study found that dietary fat content influenced the expression of *TP53INP2* expression in adipose and muscle tissues of mice [38]. This gene has been proposed to serve as a diagnostic biomarker for papillary thyroid carcinoma [39] but no study has linked its expression to cancer risk. Obesity has been convincingly correlated with breast cancer risk in numerous studies, although the relationship is complex and involves additional modifying factors [40,41]. Obesity has been associated with excess risk for breast cancer among postmenopausal women [42–46], while in pre-menopausal women, obesity was associated with decreased breast cancer risk [40,43,47–49]. However, the underlying mechanisms for this association are still not fully understood. The identification of *TP53INP2/DOR* as breast cancer-related gene could provide novel insight on the mechanism for obesity-breast cancer relationship.

In the 20q11.22 region, several other genes including *MAP1LC3A*, *ITCH*, and *TRPC4AP* were associated with ER-negative breast cancer risk. *MAP1LC3A* codes for a protein that is important in the autophagy process, and was found to be expressed at higher level in breast cancer tissues than in normal tissues [50]. E3 ubiquitin ligase ITCH plays a role in erythroid and lymphoid cell differentiation and immune response regulation, and ITCH was found to be important in the cross-talk between the Wnt and Hippo pathways in breast cancer development [51]. *TRPC4AP* is involved in Ca^2+^ signaling and is part of the ubiquitin ligase complex [52,53]. It is unclear which of these genes (or their interactions) play a role in breast cancer development, but the 20q11.22 locus is worthy of further investigation. Three of the six SNPs for *TP53INP2* (rs6060047, rs11546155, and rs1205339) are also shared by the genes *MAP1LC3A* and *TRPC4AP*. It is possible that the associations in these three genes are partly generated by the overlapped SNPs, which contribute to predicted expression levels of the three genes and, possibly, to the enrichment observed at this locus.

The *HP* gene (16q22.2) is 6,491 base pairs long and codes for a 406 amino acid preprotein, which codes haptoglobin. Haptoglobin binds to hemoglobin to prevent iron loss during hemolysis. There are two allelic forms, Hp1 (83 residues) and Hp2 (142 residues), which determine 3 major phenotypes [54]. Haptoglobin genotype has been linked to cardiocerebral outcomes among diabetic patients [55]. A small study found haptoglobin phenotypic polymorphism was associated with familial breast cancer [56], but no studies have reported on the relationship between SNPs in this gene and breast cancer risk. Further larger studies could investigate the relationship between major HP genotype/phenotype (HP1-1, HP1-2, and HP2-2) and breast cancer risk.

The present study has several strengths, including its large sample size, diverse ancestry groups, a cross-replication approach, and a novel gene expression-based analysis method. The gene-level analysis method can combine eQTL SNPs in a biologically informative way to assess relationships between predicated gene expression and disease risk. Compared to SNP-based analysis, the gene-based analysis can gain power by reducing the multiple testing burden by about 100-fold and using external information on correlation between gene expression and SNPs from reference samples. In addition, this approach enables the detection of individual SNPs with weak effects on disease risk by leveraging combined effects of multiple SNPs on gene expression. For example, none of SNPs for *TP53INP2* reached traditional genome-wide significance, but their aggregated effect via *TP53INP2* expression was genome-wide significant. The gene-based method (MetaXcan) that we employed is an extension of the gene expression-based method (PrediXcan) [29] and allows the use of SNP-level summary statistics without the need to access individual-level genotype data [30]. The MetaXcan method has been shown to produce PrediXcan results accurately, and it is robust to ancestry mismatches between studies and reference/training populations [30]. With this property, we were able to use summary statistics from the GAME-ON consortium for external replication.

Several limitations should be considered when interpreting the study findings. The gene expression-based association method relies on accurate prediction of gene transcript level from genotypes, i.e. identification of eQTLs, but eQTL identification depends on sample size of eQTL studies as well as tissue types. In the current study, we used the transcriptome prediction model that was developed using 922 RNA-seq samples from whole blood and genotype data [31]. Although it has been shown that models developed in whole blood were still useful for understanding diseases that affect other primary tissues [29], we expect there to be a loss of power when studying non-blood diseases using whole blood eQTL data. As a sensitivity analysis, we performed the MetaXcan analysis using the prediction model from breast tissues of 183 donors of multiple ethnicities (http://www.gtexportal.org). Only 4,308 genes had breast tissue specific eQTLs, and no eQTL was available for *TP53INP2*, perhaps due to the small sample size. We found that *DHODH* (*P* = 3.61×10^−5^), *ITCH* (*P* = 1.23×10^−4^), and *TRPC4AP* (*P* = 7.7x10^-4^) were among the top genes associated with ER-negative breast cancer risk, and *TRPC4AP* (*P* = 1.68×10^−5^) and *DHODH* (*P* = 1.12×10^−4^) among the top genes associated with the overall breast cancer risk using breast tissue eQTLs. In the enrichment analysis, we found that 7 (8.2%) out of 85 genes that are close to known breast cancer susceptibility loci identified in previous GWAS were associated with ER-negative breast cancer and 6 (7.1%) genes were associated with overall breast cancer risk; by contrast, of the 4223 genes away from previous GWAS loci, 199 (4.7%) genes were associated with ER-negative breast cancer and 212 (5.0%) genes were associated with overall breast cancer risk. Here, we have to consider the balance between tissue relevance and sample size in eQTL studies. Further investigations based on large, reliable eQTL datasets are desirable. In future studies, we will seek out larger samples of multi-ethnic breast tissue as training data to construct improved prediction models of gene expression and further investigate trans-ethnic associations for breast cancer.

In conclusion, our study identified *TP53INP2* and several other genes in the 20q11.22 region as potential susceptibility genes for ER-negative breast cancer using a novel gene-based analysis method that incorporates genetically determined gene expression. We demonstrated this gene-based method increases statistical power and may be helpful in searching for causal variants. Future studies need to determine whether the *TP53INP2* gene is a true susceptibility gene for breast cancer and what are the underlying mechanisms for its association with ER-negative breast cancer.

## Materials and methods

### Study samples

The study was approved by the Institutional Review Board of the University of Chicago. The Epidemiology and Genomic Research Program within the National Cancer Institute launched a Challenge at the end of 2015 to inspire novel cross-disciplinary approaches to more fully decipher the genomic basis of breast cancer, called "Up For A Challenge (U4C)–Stimulating Innovation in Breast Cancer Genetic Epidemiology”. Several data sets were gathered and made available for use in dbGap (https://www.ncbi.nlm.nih.gov/gap). Our study has two phases; the discovery phase included five U4C GWAS datasets (**Table 6**). Here, we refer them collectively as “U4C” data. These data were collected from three distinct ancestry groups. The BPC3 [16,18] and CGEMS study [15,20] were conducted in women of European ancestry. The ROOT [17] and AABC study [57] consisted of women of African ancestry. The SBCGS study was conducted in Chinese population [19]. For the analysis of overall breast cancer risk, we used four GWAS datasets: AABC, CGEMS, ROOT, and SBCGS. For the analysis of ER-negative breast cancer risk, we used datasets from AABC, BPC3, ROOT, and SBCGS. All these dbGap datasets included imputed genotype data that were inferred based on reference haplotypes from the 1000 Genomes project.

**Table 6:** dbGaP datasets used in the our gene level expression-based GWAS analysis.

Accession Number	Study Name	Acronym	Breast Cancer Phenotype	Population
phs000851	African American Breast Cancer GWAS	AABC	3016 cases, 988 ER- cases, 2745 controls	African American
phs000812	Breast and Prostate Cancer Cohort Consortium GWAS	BPC3	1998 ER- cases, 3263 controls	European American
phs000147	Cancer Genetic Markers of Susceptibility Breast Cancer GWAS	CGEMS	1142 cases, 1145 controls	European American
phs000383	GWAS of Breast Cancer in the African Diaspora	ROOT	1657 cases, 403 ER- cases, 2029 controls	African American, African, African Barbadian
phs000799	Shanghai Breast Cancer Genetic Study	SBCGS	2790 cases, 490 ER- cases, and 2176 controls	Asian (Chinese)

In the replication phase, we used the summary results from the meta-analysis of 11 breast cancer GWASs in the GAME-ON consortium (http://gameon.dfci.harvard.edu). All participants were of European ancestry. The overall breast cancer analysis included 16,003 cases and 41,335 controls from 11 GWAS studies; The ER-negative breast cancer analysis included 4939 cases and 13128 controls from 7 GWAS studies. The dataset from one study (BPC3; all ER-negative cases) in GAME-ON consortium was also included the U4C datasets. Because only meta-analysis results were available from GAME-ON, we removed the BPC3 data from “U4C” dataset when we compared replication performance to avoid duplicate counting.

### Statistical analysis

Our gene level expression-based association analysis consists of three main steps. First, we conducted SNP-level genome-wide association tests and calculated summary statistics such log odds ratios and their standard errors. We used logistic regression model adjusting for eigenvectors from the principal component analysis and related covariates such as age. Genotypes were coded by an additive genetic model. Eigenvectors in principal component analysis were calculated using the method smartPCA, which is implemented in the software EIGENSOFT version 6.0.1 [58]. For the ROOT dataset, we adjusted for age, study sites, and the top 4 eigenvectors. For the AABC dataset, we adjusted for age, study sites, and top 10 eigenvectors. For CGEMS and SBCGS, we adjusted for age and the top three or two eigenvectors, respectively. The number of eigenvectors we adjusted for was chosen according to published papers from these GWASs [17,57], as well as their association with case-control status. The logistic regression models were fit using software Mach2dat (http://www.unc.edu/~yunmli/software.html) or SNPtest [59], depending on format of the datasets; the Mach2dat software was used for CGEMS and SBCGS and SNPtest was used for ROOT and AABC. For BPC3, the GWAS summary statistics for ER-negative breast cancer have been pre-calculated in the dbGap release, so we used them directly.

Second, we applied the gene level association method, MetaXcan [30] (https://github.com/hakyimlab/MetaXcan), to each of the datasets listed in **Table 6**. MetaXcan is an extension of the method PrediXcan [29], which uses an additive genetic model to estimate the component of gene expression determined by an individual’s genetic profile and then identifies likely causal genes by computing the correlations between genetically predicted gene expression levels and disease phenotypes. MetaXcan infers the results of PrediXcan using summary statistics from GWAS, which are much more readily accessible than individual level data. In our study, as input files for MetaXcan, we used summary statistics from SNP-based analysis of each dataset obtained in step one. In addition, we used the whole blood genetic prediction model of transcriptome levels trained in the DGN data [31], which can be downloaded from http://predictdb.hakyimlab.orghttps://s3.amazonaws.com/predictdb/DGN-HapMap-2015/. The DGN data provides a large reference sample of 922 individuals with both genome-wide genotype data and RNA sequencing data. The model trained in the DGN data can be useful in estimating gene expression levels and has been successfully applied to the Wellcome Trust Case Control Consortium (WTCCC) data in identifying genes associated with five complex diseases [29]. The DGN prediction model includes a) weights for predicting gene expression using genotypes and b) covariance of the SNPs that takes into account linkage disequilibrium. We tested the association between predicted expression levels of 11,536 genes for each of the two phenotypes, overall and ER-negative breast cancer risk, using the MetaXcan software. To construct the prediction model of expression levels using the DGN data, MetaXcan used SNPs with minor allele frequencies (MAFs) >0.05. When MetaXcan was applied to the breast cancer GWAS data, only SNPs with MAFs >0.05 were used. We also looked up genes within 250 kb of the 93 breast cancer susceptibility loci identified in previous GWAS [2–4,17,32].

Third, we conducted meta-analysis to combine results from MetaXcan analyses for different datasets. The method described by Willer et al. with sample size as meta-analysis weight [60] was used. We also conducted SNP-level meta-analysis using a fixed effect model, as implemented in the software METAL (http://genome.sph.umich.edu/wiki/METAL). False discovery rates were calculated using the Benjamini and Hochberg method [61].

For genes identified in the discovery phase using the U4C datasets, we conducted replication analysis using GAME-ON summary results using the same methods described above. For each top variant and gene identified in this study, we used HaploReg [33] and USCS Genome Browser to explore functional annotations of noncoding variants. Chromatin states (promoters and enhancers), variant effect on regulatory motifs, and protein binding sites were assessed from available data from the ENCODE [62] and Roadmap Epigenomics Consortium [63]. Data from normal mammary epithelial cells (HMEC, MYO, vMHEC) were emphasized.

## Supporting information

S1 Table HP-related SNPs and their association with breast cancer risk. (DOCX)

S2 Table GAME-ON replication for SNPs related to the HP gene. (DOCX)

## References

[1] Torre LA, Bray F, Siegel RL, Ferlay J, Lortet-Tieulent J, et al (2015) Global cancer statistics, 2012. CA Cancer J Clin 65: 87–108. doi: 10.3322/caac.21262 25651787 10.3322/caac.21262

[2] Michailidou K, Beesley J, Lindstrom S, Canisius S, Dennis J, et al (2015) Genome-wide association analysis of more than 120,000 individuals identifies 15 new susceptibility loci for breast cancer. Nat Genet 47: 373–380. doi: 10.1038/ng.3242 25751625 10.1038/ng.3242 PMC4549775

[3] Garcia-Closas M, Couch FJ, Lindstrom S, Michailidou K, Schmidt MK, et al (2013) Genome-wide association studies identify four ER negative-specific breast cancer risk loci. Nat Genet 45: 392–398, 398e391-392. doi: 10.1038/ng.2561 23535733 10.1038/ng.2561 PMC3771695

[4] Michailidou K, Hall P, Gonzalez-Neira A, Ghoussaini M, Dennis J, et al (2013) Large-scale genotyping identifies 41 new loci associated with breast cancer risk. Nat Genet 45: 353–361, 361e351-352. doi: 10.1038/ng.2563 23535729 10.1038/ng.2563 PMC3771688

[5] Easton DF, Pooley KA, Dunning AM, Pharoah PD, Thompson D, et al (2007) Genome-wide association study identifies novel breast cancer susceptibility loci. Nature 447: 1087–1093. doi: 10.1038/nature05887 17529967 10.1038/nature05887 PMC2714974

[6] Stacey SN, Manolescu A, Sulem P, Rafnar T, Gudmundsson J, et al (2007) Common variants on chromosomes 2q35 and 16q12 confer susceptibility to estrogen receptor-positive breast cancer. Nature Genetics 39: 865–869. doi: 10.1038/ng2064 17529974 10.1038/ng2064

[7] Stacey SN, Manolescu A, Sulem P, Thorlacius S, Gudjonsson SA, et al (2008) Common variants on chromosome 5p12 confer susceptibility to estrogen receptor-positive breast cancer. Nature Genetics 40: 703–706. doi: 10.1038/ng.131 18438407 10.1038/ng.131

[8] Ahmed S, Thomas G, Ghoussaini M, Healey CS, Humphreys MK, et al (2009) Newly discovered breast cancer susceptibility loci on 3p24 and 17q23.2. Nature Genetics 41: 585–590. doi: 10.1038/ng.354 19330027 10.1038/ng.354 PMC2748125

[9] Thomas G, Jacobs KB, Kraft P, Yeager M, Wacholder S, et al (2009) A multistage genome-wide association study in breast cancer identifies two new risk alleles at 1p11.2 and 14q24.1 (RAD51L1). Nature Genetics 41: 579–584. doi: 10.1038/ng.353 19330030 10.1038/ng.353 PMC2928646

[10] Turnbull C, Ahmed S, Morrison J, Pernet D, Renwick A, et al (2010) Genome-wide association study identifies five new breast cancer susceptibility loci. Nature Genetics 42: 504–507. doi: 10.1038/ng.586 20453838 10.1038/ng.586 PMC3632836

[11] Antoniou AC, Wang X, Fredericksen ZS, McGuffog L, Tarrell R, et al (2010) A locus on 19p13 modifies risk of breast cancer in BRCA1 mutation carriers and is associated with hormone receptor-negative breast cancer in the general population. Nature Genetics 42: 885–892. doi: 10.1038/ng.669 20852631 10.1038/ng.669 PMC3130795

[12] Fletcher O, Johnson N, Orr N, Hosking FJ, Gibson LJ, et al (2011) Novel breast cancer susceptibility locus at 9q31.2: results of a genome-wide association study. Journal of the National Cancer Institute 103: 425–435. doi: 10.1093/jnci/djq563 21263130 10.1093/jnci/djq563

[13] Ghoussaini M, Fletcher O, Michailidou K, Turnbull C, Schmidt MK, et al (2012) Genome-wide association analysis identifies three new breast cancer susceptibility loci. Nature Genetics 44: 312–318. doi: 10.1038/ng.1049 22267197 10.1038/ng.1049 PMC3653403

[14] Bojesen SE, Pooley KA, Johnatty SE, Beesley J, Michailidou K, et al (2013) Multiple independent variants at the TERT locus are associated with telomere length and risks of breast and ovarian cancer. Nature Genetics 45: 371–384, 384e371-372. doi: 10.1038/ng.2566 23535731 10.1038/ng.2566 PMC3670748

[15] Haiman CA, Chen GK, Vachon CM, Canzian F, Dunning A, et al (2011) A common variant at the TERT-CLPTM1L locus is associated with estrogen receptor-negative breast cancer. Nat Genet 43: 1210–1214. doi: 10.1038/ng.985 22037553 10.1038/ng.985 PMC3279120

[16] Schumacher FR, Berndt SI, Siddiq A, Jacobs KB, Wang Z, et al (2011) Genome-wide association study identifies new prostate cancer susceptibility loci. Human molecular genetics 20: 3867–3875. doi: 10.1093/hmg/ddr295 21743057 10.1093/hmg/ddr295 PMC3168287

[17] Huo D, Feng Y, Haddad S, Zheng Y, Yao S, et al (2016) Genome-wide association studies in women of African ancestry identified 3q26.21 as a novel susceptibility locus for oestrogen receptor negative breast cancer. Human molecular genetics 25: 4835–4846. doi: 10.1093/hmg/ddw305 28171663 10.1093/hmg/ddw305 PMC5975608

[18] Siddiq A, Couch FJ, Chen GK, Lindstrom S, Eccles D, et al (2012) A meta-analysis of genome-wide association studies of breast cancer identifies two novel susceptibility loci at 6q14 and 20q11. Human molecular genetics 21: 5373–5384. doi: 10.1093/hmg/dds381 22976474 10.1093/hmg/dds381 PMC3510753

[19] Zheng W, Long J, Gao YT, Li C, Zheng Y, et al (2009) Genome-wide association study identifies a new breast cancer susceptibility locus at 6q25.1. Nat Genet 41: 324–328. doi: 10.1038/ng.318 19219042 10.1038/ng.318 PMC2754845

[20] Hunter DJ, Kraft P, Jacobs KB, Cox DG, Yeager M, et al (2007) A genome-wide association study identifies alleles in FGFR2 associated with risk of sporadic postmenopausal breast cancer. Nat Genet 39: 870–874. 17529973 10.1038/ng2075 PMC3493132

[21] Carey LA, Perou CM, Livasy CA, Dressler LG, Cowan D, et al (2006) Race, breast cancer subtypes, and survival in the Carolina Breast Cancer Study. JAMA 295: 2492–2502. doi: 10.1001/jama.295.21.2492 16757721 10.1001/jama.295.21.2492

[22] Huo D, Ikpatt F, Khramtsov A, Dangou JM, Nanda R, et al (2009) Population differences in breast cancer: survey in indigenous african women reveals over-representation of triple-negative breast cancer. J Clin Oncol 27: 4515–4521. doi: 10.1200/JCO.2008.19.6873 19704069 10.1200/JCO.2008.19.6873 PMC2754904

[23] Eng A, McCormack V, dos-Santos-Silva I (2014) Receptor-defined subtypes of breast cancer in indigenous populations in Africa: a systematic review and meta-analysis. PLoS Med 11: e1001720 doi: 10.1371/journal.pmed.1001720 25202974 10.1371/journal.pmed.1001720 PMC4159229

[24] Huo D, Zheng Y, Ogundiran TO, Adebamowo C, Nathanson KL, et al (2012) Evaluation of 19 susceptibility loci of breast cancer in women of African ancestry. Carcinogenesis 33: 835–840. doi: 10.1093/carcin/bgs093 22357627 10.1093/carcin/bgs093 PMC3324445

[25] Feng Y, Stram DO, Rhie SK, Millikan RC, Ambrosone CB, et al (2014) A comprehensive examination of breast cancer risk loci in African American women. Human molecular genetics 23: 5518–5526. doi: 10.1093/hmg/ddu252 24852375 10.1093/hmg/ddu252 PMC4168823

[26] DIAbetes Genetics Replication Meta-analysis C, Asian Genetic Epidemiology Network Type 2 Diabetes C, South Asian Type 2 Diabetes C, Mexican American Type 2 Diabetes C, Type 2 Diabetes Genetic Exploration by Nex-generation sequencing in muylti-Ethnic Samples C, et al (2014) Genome-wide trans-ancestry meta-analysis provides insight into the genetic architecture of type 2 diabetes susceptibility. Nat Genet 46: 234–244. doi: 10.1038/ng.2897 24509480 10.1038/ng.2897 PMC3969612

[27] Nicolae DL, Gamazon E, Zhang W, Duan S, Dolan ME, et al (2010) Trait-associated SNPs are more likely to be eQTLs: annotation to enhance discovery from GWAS. PLoS Genet 6: e1000888 doi: 10.1371/journal.pgen.1000888 20369019 10.1371/journal.pgen.1000888 PMC2848547

[28] Gusev A, Lee SH, Trynka G, Finucane H, Vilhjalmsson BJ, et al (2014) Partitioning heritability of regulatory and cell-type-specific variants across 11 common diseases. Am J Hum Genet 95: 535–552. doi: 10.1016/j.ajhg.2014.10.004 25439723 10.1016/j.ajhg.2014.10.004 PMC4225595

[29] Gamazon ER, Wheeler HE, Shah KP, Mozaffari SV, Aquino-Michaels K, et al (2015) A gene-based association method for mapping traits using reference transcriptome data. Nat Genet 47: 1091–1098. doi: 10.1038/ng.3367 26258848 10.1038/ng.3367 PMC4552594

[30] Barbeira A, Shah KP, Torres JM, Wheeler HE, Torstenson ES, et al. (2016) MetaXcan: Summary Statistics Based Gene-Level Association Method Infers Accurate PrediXcan Results. BioRxiv: doi: https://doi.org/10.1101/045260

[31] Battle A, Mostafavi S, Zhu X, Potash JB, Weissman MM, et al (2014) Characterizing the genetic basis of transcriptome diversity through RNA-sequencing of 922 individuals. Genome Res 24: 14–24. doi: 10.1101/gr.155192.113 24092820 10.1101/gr.155192.113 PMC3875855

[32] Mavaddat N, Pharoah PD, Michailidou K, Tyrer J, Brook MN, et al (2015) Prediction of breast cancer risk based on profiling with common genetic variants. J Natl Cancer Inst 107. 10.1093/jnci/djv036 PMC4754625 25855707

[33] Ward LD, Kellis M (2012) HaploReg: a resource for exploring chromatin states, conservation, and regulatory motif alterations within sets of genetically linked variants. Nucleic Acids Res 40: D930–934. doi: 10.1093/nar/gkr917 22064851 10.1093/nar/gkr917 PMC3245002

[34] Bhattacharya A, Schmitz U, Raatz Y, Schonherr M, Kottek T, et al (2015) miR-638 promotes melanoma metastasis and protects melanoma cells from apoptosis and autophagy. Oncotarget 6: 2966–2980. 25650662 10.18632/oncotarget.3070 PMC4413631

[35] Malkin D, Li FP, Strong LC, Fraumeni JF Jr., Nelson CE, et al (1990) Germ line p53 mutations in a familial syndrome of breast cancer, sarcomas, and other neoplasms. Science 250: 1233–1238. 1978757 10.1126/science.1978757

[36] Cancer Genome Atlas Network (2012) Comprehensive molecular portraits of human breast tumours. Nature 490: 61–70. doi: 10.1038/nature11412 23000897 10.1038/nature11412 PMC3465532

[37] Kruse R, Vind BF, Petersson SJ, Kristensen JM, Hojlund K (2015) Markers of autophagy are adapted to hyperglycaemia in skeletal muscle in type 2 diabetes. Diabetologia 58: 2087–2095. 26048236 10.1007/s00125-015-3654-0

[38] Fromm-Dornieden C, Lytovchenko O, von der Heyde S, Behnke N, Hogl S, et al (2012) Extrinsic and intrinsic regulation of DOR/TP53INP2 expression in mice: effects of dietary fat content, tissue type and sex in adipose and muscle tissues. Nutr Metab (Lond) 9: 86. 22995226 10.1186/1743-7075-9-86 PMC3497704

[39] Zhao Y, Liu X, Zhong L, He M, Chen S, et al (2015) The combined use of miRNAs and mRNAs as biomarkers for the diagnosis of papillary thyroid carcinoma. Int J Mol Med 36: 1097–1103. doi: 10.3892/ijmm.2015.2305 26252081 10.3892/ijmm.2015.2305

[40] Hunter DJ, Willett WC (1993) Diet, body size, and breast cancer. Epidemiol Rev 15: 110–132. 8405195 10.1093/oxfordjournals.epirev.a036096

[41] Ursin G, Longnecker MP, Haile RW, Greenland S (1995) A meta-analysis of body mass index and risk of premenopausal breast cancer. Epidemiology 6: 137–141. 7742399 10.1097/00001648-199503000-00009

[42] Lam PB, Vacek PM, Geller BM, Muss HB (2000) The association of increased weight, body mass index, and tissue density with the risk of breast carcinoma in Vermont. Cancer 89: 369–375. 10918168 10.1002/1097-0142(20000715)89:2<369::aid-cncr23>3.0.co;2-j

[43] Huang WY, Newman B, Millikan RC, Schell MJ, Hulka BS, et al (2000) Hormone-related factors and risk of breast cancer in relation to estrogen receptor and progesterone receptor status. Am J Epidemiol 151: 703–714. 10752798 10.1093/oxfordjournals.aje.a010265

[44] Pathak DR, Whittemore AS (1992) Combined effects of body size, parity, and menstrual events on breast cancer incidence in seven countries. Am J Epidemiol 135: 153–168. 1536132 10.1093/oxfordjournals.aje.a116268

[45] Chu SY, Lee NC, Wingo PA, Senie RT, Greenberg RS, et al (1991) The relationship between body mass and breast cancer among women enrolled in the Cancer and Steroid Hormone Study. J Clin Epidemiol 44: 1197–1206. 1941014 10.1016/0895-4356(91)90152-y

[46] Toniolo PG, Levitz M, Zeleniuch-Jacquotte A, Banerjee S, Koenig KL, et al (1995) A prospective study of endogenous estrogens and breast cancer in postmenopausal women. J Natl Cancer Inst 87: 190–197. 7707406 10.1093/jnci/87.3.190

[47] van den Brandt PA, Spiegelman D, Yaun SS, Adami HO, Beeson L, et al (2000) Pooled analysis of prospective cohort studies on height, weight, and breast cancer risk. Am J Epidemiol 152: 514–527. 10997541 10.1093/aje/152.6.514

[48] Willett WC, Browne ML, Bain C, Lipnick RJ, Stampfer MJ, et al (1985) Relative weight and risk of breast cancer among premenopausal women. Am J Epidemiol 122: 731–740. 4050766 10.1093/oxfordjournals.aje.a114156

[49] Ogundiran TO, Huo D, Adenipekun A, Campbell O, Oyesegun R, et al (2010) Case-control study of body size and breast cancer risk in Nigerian women. Am J Epidemiol 172: 682–690. doi: 10.1093/aje/kwq180 20716701 10.1093/aje/kwq180 PMC2950817

[50] Othman EQ, Kaur G, Mutee AF, Muhammad TS, Tan ML (2009) Immunohistochemical expression of MAP1LC3A and MAP1LC3B protein in breast carcinoma tissues. J Clin Lab Anal 23: 249–258. doi: 10.1002/jcla.20309 19623642 10.1002/jcla.20309 PMC6648937

[51] Lim SK, Lu SY, Kang SA, Tan HJ, Li Z, et al (2016) Wnt Signaling Promotes Breast Cancer by Blocking ITCH-Mediated Degradation of YAP/TAZ Transcriptional Coactivator WBP2. Cancer Res 76: 6278–6289. 27578003 10.1158/0008-5472.CAN-15-3537

[52] Jamal A, Swarnalatha M, Sultana S, Joshi P, Panda SK, et al (2015) The G1 phase E3 ubiquitin ligase TRUSS that gets deregulated in human cancers is a novel substrate of the S-phase E3 ubiquitin ligase Skp2. Cell Cycle 14: 2688–2700. doi: 10.1080/15384101.2015.1056946 26038816 10.1080/15384101.2015.1056946 PMC4612110

[53] Mace KE, Lussier MP, Boulay G, Terry-Powers JL, Parfrey H, et al (2010) TRUSS, TNF-R1, and TRPC ion channels synergistically reverse endoplasmic reticulum Ca2+ storage reduction in response to m1 muscarinic acetylcholine receptor signaling. J Cell Physiol 225: 444–453. doi: 10.1002/jcp.22221 20458742 10.1002/jcp.22221 PMC4447216

[54] Langlois MR, Delanghe JR (1996) Biological and clinical significance of haptoglobin polymorphism in humans. Clin Chem 42: 1589–1600. 8855140

[55] Vardi M, Blum S, Levy AP (2012) Haptoglobin genotype and cardiovascular outcomes in diabetes mellitus—natural history of the disease and the effect of vitamin E treatment. Meta-analysis of the medical literature. Eur J Intern Med 23: 628–632. doi: 10.1016/j.ejim.2012.04.009 22939808 10.1016/j.ejim.2012.04.009 PMC3600118

[56] Awadallah SM, Atoum MF (2004) Haptoglobin polymorphism in breast cancer patients form Jordan. Clin Chim Acta 341: 17–21. doi: 10.1016/j.cccn.2003.10.032 14967153 10.1016/j.cccn.2003.10.032

[57] Chen F, Chen GK, Stram DO, Millikan RC, Ambrosone CB, et al (2013) A genome-wide association study of breast cancer in women of African ancestry. Hum Genet 132: 39–48. doi: 10.1007/s00439-012-1214-y 22923054 10.1007/s00439-012-1214-y PMC3749077

[58] Patterson N, Price AL, Reich D (2006) Population structure and eigenanalysis. PLoS Genet 2: e190 doi: 10.1371/journal.pgen.0020190 17194218 10.1371/journal.pgen.0020190 PMC1713260

[59] Marchini J, Howie B (2010) Genotype imputation for genome-wide association studies. Nat Rev Genet 11: 499–511. 20517342 10.1038/nrg2796

[60] Willer CJ, Li Y, Abecasis GR (2010) METAL: fast and efficient meta-analysis of genomewide association scans. Bioinformatics 26: 2190–2191. doi: 10.1093/bioinformatics/btq340 20616382 10.1093/bioinformatics/btq340 PMC2922887

[61] Benjamini Y, Hochberg Y (1995) Controlling the false discovery rate—a practical and powerful approach to multiple testing. J Royal Stat Soc, Series B 57: 289–300.

[62] Encode Project Consortium (2011) A user's guide to the encyclopedia of DNA elements (ENCODE). PLoS Biol 9: e1001046 doi: 10.1371/journal.pbio.1001046 21526222 10.1371/journal.pbio.1001046 PMC3079585

[63] Roadmap Epigenomics C, Kundaje A, Meuleman W, Ernst J, Bilenky M, et al (2015) Integrative analysis of 111 reference human epigenomes. Nature 518: 317–330. doi: 10.1038/nature14248 25693563 10.1038/nature14248 PMC4530010

