# Supplementary material for: Trans-ethnic predicted expression genome-wide association analysis identifies a gene for estrogen receptor-negative breast cancer
Source: PLoS Genet. 2017 Sep 28;13(9):e1006727. doi: 10.1371/journal.pgen.1006727 (PMC5619687; doi:10.1371/journal.pgen.1006727)
Supplement: S1 Table — (DOCX) [file pgen.1006727.s001.docx]

**S1 Table**. HP-related SNPs and their association with breast cancer risk

|  | Pos. at chr16* | Test/ref allele |  | Overall | | ER-negative | |
| --- | --- | --- | --- | --- | --- | --- | --- |
| SNP |  |  | Study | OR (95% CI) | P | OR (95% CI) | P |
| rs236009 | 71251795 | C/T | CGEMS | 0.91 (0.81-1.03) | 0.13 |  | . |
|  |  |  | BPC3 |  | . | 1.00 (0.91-1.08) | 0.92 |
|  |  |  | AABC | 1.00 (0.88-1.14) | 0.99 | 0.91 (0.76-1.10) | 0.34 |
|  |  |  | ROOT | 0.52 (0.11-2.40) | 0.4 | 1.05 (0.74-1.48) | 0.79 |
|  |  |  | SBCGS | 0.98 (0.88-1.08) | 0.64 | 0.91 (0.76-1.08) | 0.27 |
|  |  |  | meta | 0.96 (0.90-1.03) | 0.24 | 0.97 (0.91-1.04) | 0.41 |
| rs236008 | 71254941 | T/C | CGEMS | 0.91 (0.81-1.03) | 0.13 |  | . |
|  |  |  | BPC3 |  | . | 1.00 (0.91-1.08) | 0.92 |
|  |  |  | AABC | 1.00 (0.88-1.14) | 0.99 | 0.92 (0.76-1.10) | 0.34 |
|  |  |  | ROOT | 0.52 (0.11-2.40) | 0.4 | 1.05 (0.74-1.48) | 0.79 |
|  |  |  | SBCGS | 0.98 (0.88-1.08) | 0.63 | 0.91 (0.76-1.08) | 0.28 |
|  |  |  | meta | 0.96 (0.90-1.03) | 0.23 | 0.97 (0.91-1.04) | 0.41 |
| rs11645475 | 71823117 | C/T | CGEMS | 1.01 (0.75-1.36) | 0.93 |  | . |
|  |  |  | BPC3 |  | . | 1.15 (0.93-1.42) | 0.2 |
|  |  |  | AABC | 0.92 (0.62-1.36) | 0.67 | 1.03 (0.58-1.82) | 0.93 |
|  |  |  | ROOT | 0.61 (0.34-1.10) | 0.099 | 0.57 (0.24-1.37) | 0.21 |
|  |  |  | SBCGS | 0.94 (0.79-1.13) | 0.51 | 0.78 (0.55-1.11) | 0.16 |
|  |  |  | meta | 0.93 (0.81-1.07) | 0.31 | 1.01 (0.85-1.20) | 0.9 |
| rs1035559 | 72031860 | G/A | CGEMS | 0.89 (0.79-1.00) | 0.046 |  | . |
|  |  |  | BPC3 |  | . | 0.89 (0.82-0.97) | 0.01 |
|  |  |  | AABC | 0.93 (0.86-1.01) | 0.082 | 0.94 (0.84-1.06) | 0.3 |
|  |  |  | ROOT | 0.93 (0.84-1.02) | 0.14 | 0.93 (0.79-1.10) | 0.4 |
|  |  |  | SBCGS | 0.94 (0.86-1.04) | 0.23 | 0.82 (0.69-0.97) | 0.021 |
|  |  |  | meta | 0.93 (0.88-0.97) | 1.6E-03 | 0.90 (0.85-0.96) | 6.4E-04 |
| rs7195958 | 72036577 | G/A | BPC3 |  | . | 1.15 (1.06-1.25) | 1.2E-03 |
|  |  |  | AABC | 1.07 (0.99-1.16) | 0.078 | 1.04 (0.93-1.16) | 0.53 |
|  |  |  | ROOT | 1.07 (0.97-1.18) | 0.18 | 1.05 (0.89-1.24) | 0.57 |
|  |  |  | SBCGS | 1.06 (0.97-1.16) | 0.22 | 1.20 (1.02-1.42) | 0.031 |
|  |  |  | meta | 1.07 (1.01-1.12) | 0.012 | 1.11 (1.05-1.18) | 4.1E-04 |
| rs3213422 | 72042682 | C/A | CGEMS | 1.14 (1.02-1.28) | 0.026 |  | . |
|  |  |  | BPC3 |  | . | 1.16 (1.06-1.26) | 7.6E-04 |
|  |  |  | AABC | 1.09 (1.01-1.17) | 0.036 | 1.06 (0.95-1.19) | 0.28 |
|  |  |  | ROOT | 1.13 (1.03-1.25) | 9.9E-03 | 1.14 (0.97-1.35) | 0.11 |
|  |  |  | SBCGS | 1.05 (0.96-1.15) | 0.28 | 1.17 (0.99-1.38) | 0.063 |
|  |  |  | meta | 1.10 (1.05-1.15) | 9.1E-05 | 1.13 (1.07-1.20) | 4.2E-05 |
| rs11641424 | 72066879 | T/C | CGEMS | 1.14 (0.97-1.34) | 0.12 |  | . |
|  |  |  | BPC3 |  | . | 1.22 (1.08-1.37) | 1.3E-03 |
|  |  |  | AABC | 1.29 (1.03-1.61) | 0.025 | 1.22 (0.88-1.68) | 0.23 |
|  |  |  | ROOT | 1.08 (0.75-1.55) | 0.68 | 0.99 (0.57-1.73) | 0.97 |
|  |  |  | SBCGS | 1.20 (0.91-1.58) | 0.2 | 1.20 (0.75-1.94) | 0.45 |
|  |  |  | meta | 1.18 (1.05-1.32) | 4.2E-03 | 1.21 (1.08-1.34) | 5.8E-04 |
| rs1424241 | 72078907 | A/G | CGEMS | 1.16 (0.99-1.34) | 0.06 |  | . |
|  |  |  | BPC3 |  | . | 1.23 (1.10-1.37) | 2.20E-04 |
|  |  |  | AABC | 1.04 (0.94-1.15) | 0.4 | 1.08 (0.94-1.25) | 0.26 |
|  |  |  | ROOT | 1.01 (0.89-1.14) | 0.91 | 0.91 (0.74-1.12) | 0.4 |
|  |  |  | SBCGS | 1.05 (0.88-1.26) | 0.57 | 0.98 (0.71-1.34) | 0.89 |
|  |  |  | meta | 1.05 (0.99-1.12) | 0.11 | 1.12 (1.04-1.21) | 3.9E-03 |
| rs5467 | 72088280 | T/C | CGEMS | 1.05 (0.91-1.21) | 0.51 |  | . |
|  |  |  | BPC3 |  | . | 1.19 (1.08-1.32) | 8.1E-04 |
|  |  |  | AABC | 1.16 (0.97-1.38) | 0.11 | 1.07 (0.82-1.39) | 0.61 |
|  |  |  | ROOT | 0.99 (0.73-1.33) | 0.92 | 1.00 (0.63-1.57) | 0.99 |
|  |  |  | SBCGS | 1.06 (0.89-1.26) | 0.53 | 0.98 (0.72-1.34) | 0.9 |
|  |  |  | meta | 1.07 (0.98-1.17) | 0.13 | 1.15 (1.05-1.26) | 2.2E-03 |
| rs5468 | 72088331 | G/T | CGEMS | 0.93 (0.77-1.13) | 0.48 |  | . |
|  |  |  | BPC3 |  | . | 1.15 (1.00-1.33) | 0.051 |
|  |  |  | AABC | 1.10 (0.85-1.42) | 0.48 | 1.25 (0.87-1.79) | 0.23 |
|  |  |  | ROOT | 1.17 (0.77-1.79) | 0.46 | 1.16 (0.60-2.24) | 0.66 |
|  |  |  | SBCGS | 1.36 (0.92-2.00) | 0.12 | 1.36 (0.70-2.64) | 0.36 |
|  |  |  | meta | 1.04 (0.91-1.19) | 0.53 | 1.17 (1.03-1.33) | 0.015 |
| rs8062041 | 72088964 | T/C | CGEMS | 0.87 (0.77-0.98) | 0.022 |  | . |
|  |  |  | BPC3 |  | . | 0.90 (0.82-0.98) | 0.017 |
|  |  |  | AABC | 0.91 (0.84-0.99) | 0.021 | 0.91 (0.81-1.01) | 0.08 |
|  |  |  | ROOT | 0.89 (0.81-0.99) | 0.024 | 0.87 (0.74-1.02) | 0.092 |
|  |  |  | SBCGS | 0.96 (0.88-1.06) | 0.44 | 0.88 (0.74-1.04) | 0.12 |
|  |  |  | meta | 0.91 (0.87-0.96) | 1.7E-04 | 0.89 (0.84-0.95) | 2.1E-04 |
| rs2000999 | 72108093 | A/G | CGEMS | 0.94 (0.82-1.09) | 0.4 |  | . |
|  |  |  | BPC3 |  | . | 0.83 (0.74-0.92) | 6.5E-04 |
|  |  |  | AABC | 0.93 (0.80-1.08) | 0.35 | 1.10 (0.89-1.36) | 0.39 |
|  |  |  | ROOT | 1.01 (0.83-1.22) | 0.95 | 0.95 (0.69-1.33) | 0.78 |
|  |  |  | SBCGS | 0.96 (0.88-1.06) | 0.41 | 0.87 (0.74-1.03) | 0.11 |
|  |  |  | meta | 0.96 (0.90-1.02) | 0.18 | 0.88 (0.81-0.96) | 2.1E-03 |
| rs9941087 | 72118324 | A/G | CGEMS | 0.88 (0.78-1.00) | 0.043 |  | . |
|  |  |  | BPC3 |  | . | 0.89 (0.81-0.97) | 7.2E-03 |
|  |  |  | AABC | 0.91 (0.84-0.98) | 0.018 | 0.90 (0.81-1.01) | 0.073 |
|  |  |  | ROOT | 0.90 (0.81-0.99) | 0.027 | 0.86 (0.73-1.01) | 0.067 |
|  |  |  | SBCGS | 0.97 (0.88-1.06) | 0.47 | 0.87 (0.74-1.03) | 0.1 |
|  |  |  | meta | 0.92 (0.88-0.96) | 2.9E-04 | 0.88 (0.83-0.94) | 5.7E-05 |
| rs1050362 | 72130815 | A/C | CGEMS | 0.89 (0.79-1.00) | 0.046 |  | . |
|  |  |  | BPC3 |  | . | 0.90 (0.82-0.98) | 0.016 |
|  |  |  | AABC | 0.91 (0.84-0.99) | 0.025 | 0.95 (0.85-1.08) | 0.44 |
|  |  |  | ROOT | 0.93 (0.83-1.04) | 0.2 | 0.78 (0.65-0.94) | 9.5E-03 |
|  |  |  | SBCGS | 0.97 (0.88-1.06) | 0.48 | 0.87 (0.74-1.02) | 0.083 |
|  |  |  | meta | 0.93 (0.88-0.97) | 2.1E-03 | 0.89 (0.84-0.95) | 3.2E-04 |
| rs2072142 | 72132713 | T/C | CGEMS | 0.89 (0.79-1.00) | 0.047 |  | . |
|  |  |  | BPC3 |  | . | 0.90 (0.82-0.98) | 0.016 |
|  |  |  | AABC | 0.91 (0.83-0.99) | 0.022 | 0.95 (0.84-1.07) | 0.41 |
|  |  |  | ROOT | 0.93 (0.83-1.04) | 0.2 | 0.78 (0.65-0.94) | 9.7E-03 |
|  |  |  | SBCGS | 0.97 (0.88-1.06) | 0.47 | 0.87 (0.74-1.02) | 0.083 |
|  |  |  | meta | 0.93 (0.88-0.97) | 1.9E-03 | 0.89 (0.84-0.95) | 2.9E-04 |
| rs2074627 | 72136769 | T/C | CGEMS | 0.89 (0.79-1.00) | 0.053 |  | . |
|  |  |  | BPC3 |  | . | 0.90 (0.82-0.98) | 0.016 |
|  |  |  | AABC | 0.91 (0.83-0.98) | 0.02 | 0.95 (0.84-1.07) | 0.42 |
|  |  |  | ROOT | 0.93 (0.83-1.04) | 0.18 | 0.79 (0.66-0.95) | 0.012 |
|  |  |  | SBCGS | 0.97 (0.88-1.06) | 0.47 | 0.86 (0.73-1.02) | 0.077 |
|  |  |  | meta | 0.92 (0.88-0.97) | 1.6E-03 | 0.89 (0.84-0.95) | 3.2E-04 |
| rs12325142 | 72138112 | T/G | CGEMS | 0.89 (0.79-1.00) | 0.054 |  | . |
|  |  |  | BPC3 |  | . | 0.90 (0.82-0.98) | 0.016 |
|  |  |  | AABC | 0.90 (0.83-0.98) | 0.018 | 0.95 (0.84-1.07) | 0.42 |
|  |  |  | ROOT | 0.93 (0.83-1.04) | 0.19 | 0.79 (0.66-0.95) | 0.012 |
|  |  |  | SBCGS | 0.97 (0.88-1.06) | 0.46 | 0.86 (0.73-1.02) | 0.077 |
|  |  |  | meta | 0.92 (0.88-0.97) | 1.6E-03 | 0.89 (0.84-0.95) | 3.3E-04 |
| rs2074626 | 72139184 | A/C | CGEMS | 0.89 (0.79-1.00) | 0.054 |  | . |
|  |  |  | BPC3 |  | . | 0.90 (0.82-0.98) | 0.018 |
|  |  |  | AABC | 0.91 (0.83-0.99) | 0.022 | 0.95 (0.84-1.07) | 0.42 |
|  |  |  | ROOT | 0.93 (0.83-1.04) | 0.21 | 0.80 (0.66-0.96) | 0.015 |
|  |  |  | SBCGS | 0.97 (0.88-1.06) | 0.46 | 0.86 (0.73-1.02) | 0.076 |
|  |  |  | meta | 0.93 (0.88-0.97) | 2.1E-03 | 0.89 (0.84-0.95) | 3.9E-04 |
| rs6499560 | 72147666 | C/A | CGEMS | 0.89 (0.79-1.00) | 0.054 |  | . |
|  |  |  | BPC3 |  | . | 0.90 (0.82-0.98) | 0.017 |
|  |  |  | AABC | 0.89 (0.82-0.97) | 7.0E-03 | 0.96 (0.85-1.08) | 0.51 |
|  |  |  | ROOT | 0.94 (0.84-1.04) | 0.23 | 0.77 (0.64-0.92) | 5.1E-03 |
|  |  |  | SBCGS | 0.96 (0.88-1.06) | 0.43 | 0.87 (0.74-1.02) | 0.082 |
|  |  |  | meta | 0.92 (0.88-0.97) | 8.5E-04 | 0.89 (0.84-0.95) | 3.5E-04 |
| rs17604349 | 72210865 | A/G | CGEMS | 1.10 (0.94-1.27) | 0.23 |  | . |
|  |  |  | BPC3 |  | . | 1.08 (0.97-1.20) | 0.15 |
|  |  |  | AABC | 1.22 (1.03-1.45) | 0.023 | 1.19 (0.93-1.53) | 0.17 |
|  |  |  | ROOT | 1.17 (0.89-1.53) | 0.27 | 1.23 (0.80-1.90) | 0.34 |
|  |  |  | SBCGS | 1.07 (0.89-1.29) | 0.44 | 1.14 (0.84-1.55) | 0.41 |
|  |  |  | meta | 1.13 (1.03-1.24) | 7.9E-03 | 1.11 (1.01-1.21) | 0.029 |

*NCBI 37

None of the tests for heterogeneity across studies was significant.

OR, odds ratio; CI, confidence intervals; ER, estrogen receptor

**S2 Table.** GAME-ON replication for SNPs related to the HP gene

|  | Test/ref allele | Study phase | Overall | | ER-negative* | |
| --- | --- | --- | --- | --- | --- | --- |
| SNP |  |  | OR (95% CI) | P | OR (95% CI) | P |
| rs1035559 | G/A | U4C | 0.93 (0.88-0.97) | 1.60E-03 | 0.91 (0.84-0.99) | 0.024 |
| rs1035559 | G/A | GAME-ON | 0.97 (0.94-1.01) | 0.1 | 0.95 (0.90-1.01) | 0.079 |
| rs1050362 | A/C | U4C | 0.93 (0.88-0.97) | 2.10E-03 | 0.89 (0.82-0.97) | 7.70E-03 |
| rs1050362 | A/C | GAME-ON | 0.97 (0.94-1.01) | 0.11 | 0.95 (0.90-1.01) | 0.075 |
| rs11641424 | T/C | U4C | 1.18 (1.05-1.32) | 4.20E-03 | 1.17 (0.92-1.49) | 0.21 |
| rs11641424 | T/C | GAME-ON | 1.01 (0.97-1.06) | 0.62 | 1.04 (0.97-1.12) | 0.25 |
| rs11645475 | C/T | U4C | 0.93 (0.81-1.07) | 0.31 | 0.81 (0.61-1.07) | 0.14 |
| rs11645475 | C/T | GAME-ON | 1.09 (1.00-1.19) | 0.057 | 1.13 (0.96-1.33) | 0.14 |
| rs12325142 | T/G | U4C | 0.92 (0.88-0.97) | 1.60E-03 | 0.89 (0.82-0.97) | 7.50E-03 |
| rs12325142 | T/G | GAME-ON | 0.97 (0.94-1.01) | 0.12 | 0.95 (0.90-1.00) | 0.071 |
| rs1424241 | A/G | U4C | 1.05 (0.99-1.12) | 0.11 | 1.02 (0.92-1.14) | 0.7 |
| rs1424241 | A/G | GAME-ON | 1.02 (0.98-1.07) | 0.27 | 1.04 (0.97-1.11) | 0.24 |
| rs17604349 | A/G | U4C | 1.13 (1.03-1.24) | 7.90E-03 | 1.18 (0.99-1.41) | 0.066 |
| rs17604349 | A/G | GAME-ON | 1.04 (0.99-1.08) | 0.092 | 1.02 (0.96-1.09) | 0.53 |
| rs2000999 | A/G | U4C | 0.96 (0.90-1.02) | 0.18 | 0.95 (0.84-1.08) | 0.43 |
| rs2000999 | A/G | GAME-ON | 0.99 (0.95-1.04) | 0.69 | 0.94 (0.87-1.00) | 0.054 |
| rs2072142 | T/C | U4C | 0.93 (0.88-0.97) | 1.90E-03 | 0.89 (0.82-0.97) | 6.80E-03 |
| rs2072142 | T/C | GAME-ON | 0.97 (0.94-1.01) | 0.1 | 0.95 (0.90-1.00) | 0.071 |
| rs2074626 | A/C | U4C | 0.93 (0.88-0.97) | 2.10E-03 | 0.89 (0.82-0.97) | 8.20E-03 |
| rs2074626 | A/C | GAME-ON | 0.97 (0.94-1.01) | 0.12 | 0.95 (0.90-1.01) | 0.085 |
| rs2074627 | T/C | U4C | 0.92 (0.88-0.97) | 1.60E-03 | 0.89 (0.82-0.97) | 7.30E-03 |
| rs2074627 | T/C | GAME-ON | 0.97 (0.94-1.01) | 0.12 | 0.95 (0.90-1.00) | 0.072 |
| rs236008 | T/C | U4C | 0.96 (0.90-1.03) | 0.23 | 0.93 (0.82-1.04) | 0.21 |
| rs236008 | T/C | GAME-ON | 0.98 (0.95-1.02) | 0.31 | 0.98 (0.93-1.03) | 0.49 |
| rs236009 | C/T | U4C | 0.96 (0.90-1.03) | 0.24 | 0.93 (0.82-1.04) | 0.2 |
| rs236009 | C/T | GAME-ON | 0.98 (0.95-1.02) | 0.31 | 0.98 (0.93-1.03) | 0.46 |
| rs3213422 | C/A | U4C | 1.10 (1.05-1.15) | 9.10E-05 | 1.11 (1.02-1.20) | 0.014 |
| rs3213422 | C/A | GAME-ON | 1.02 (0.99-1.06) | 0.18 | 1.07 (1.02-1.13) | 0.01 |
| rs5467 | T/C | U4C | 1.07 (0.98-1.17) | 0.13 | 1.03 (0.85-1.23) | 0.78 |
| rs5467 | T/C | GAME-ON | 1.02 (0.98-1.06) | 0.36 | 1.03 (0.97-1.10) | 0.34 |
| rs5468 | G/T | U4C | 1.04 (0.91-1.19) | 0.53 | 1.25 (0.94-1.66) | 0.13 |
| rs5468 | G/T | GAME-ON | 1.02 (0.96-1.09) | 0.47 | 1.05 (0.94-1.18) | 0.36 |
| rs6499560 | C/A | U4C | 0.92 (0.88-0.97) | 8.50E-04 | 0.89 (0.82-0.97) | 7.50E-03 |
| rs6499560 | C/A | GAME-ON | 0.97 (0.94-1.01) | 0.1 | 0.95 (0.90-1.01) | 0.093 |
| rs7195958 | G/A | U4C | 1.07 (1.01-1.12) | 0.012 | 1.08 (0.99-1.17) | 0.075 |
| rs7195958 | G/A | GAME-ON | 1.02 (0.99-1.06) | 0.18 | 1.07 (1.02-1.13) | 0.011 |
| rs8062041 | T/C | U4C | 0.91 (0.87-0.96) | 1.70E-04 | 0.89 (0.82-0.96) | 4.50E-03 |
| rs8062041 | T/C | GAME-ON | 0.97 (0.94-1.01) | 0.13 | 0.95 (0.90-1.01) | 0.089 |
| rs9941087 | A/G | U4C | 0.92 (0.88-0.96) | 2.90E-04 | 0.88 (0.82-0.96) | 2.70E-03 |
| rs9941087 | A/G | GAME-ON | 0.97 (0.94-1.01) | 0.11 | 0.95 (0.90-1.00) | 0.053 |

*The overlapping study (BPC3) was removed from the meta-analysis in the discovery phase (U4C).

OR, odds ratio; CI, confidence intervals; ER, estrogen receptor
