# Supplementary material for: Trans-ethnic predicted expression genome-wide association analysis identifies a gene for estrogen receptor-negative breast cancer
Source: PLoS Genet. 2017 Sep 28;13(9):e1006727. doi: 10.1371/journal.pgen.1006727 (PMC5619687; doi:10.1371/journal.pgen.1006727)
Supplement: S2 Table — (DOCX) [file pgen.1006727.s002.docx]

**S2 Table.** GAME-ON replication for SNPs related to the HP gene

|  | Test/ref allele | Study phase | Overall | | ER-negative* | |
| --- | --- | --- | --- | --- | --- | --- |
| SNP |  |  | OR (95% CI) | P | OR (95% CI) | P |
| rs1035559 | G/A | U4C | 0.93 (0.88-0.97) | 1.60E-03 | 0.91 (0.84-0.99) | 0.024 |
| rs1035559 | G/A | GAME-ON | 0.97 (0.94-1.01) | 0.1 | 0.95 (0.90-1.01) | 0.079 |
| rs1050362 | A/C | U4C | 0.93 (0.88-0.97) | 2.10E-03 | 0.89 (0.82-0.97) | 7.70E-03 |
| rs1050362 | A/C | GAME-ON | 0.97 (0.94-1.01) | 0.11 | 0.95 (0.90-1.01) | 0.075 |
| rs11641424 | T/C | U4C | 1.18 (1.05-1.32) | 4.20E-03 | 1.17 (0.92-1.49) | 0.21 |
| rs11641424 | T/C | GAME-ON | 1.01 (0.97-1.06) | 0.62 | 1.04 (0.97-1.12) | 0.25 |
| rs11645475 | C/T | U4C | 0.93 (0.81-1.07) | 0.31 | 0.81 (0.61-1.07) | 0.14 |
| rs11645475 | C/T | GAME-ON | 1.09 (1.00-1.19) | 0.057 | 1.13 (0.96-1.33) | 0.14 |
| rs12325142 | T/G | U4C | 0.92 (0.88-0.97) | 1.60E-03 | 0.89 (0.82-0.97) | 7.50E-03 |
| rs12325142 | T/G | GAME-ON | 0.97 (0.94-1.01) | 0.12 | 0.95 (0.90-1.00) | 0.071 |
| rs1424241 | A/G | U4C | 1.05 (0.99-1.12) | 0.11 | 1.02 (0.92-1.14) | 0.7 |
| rs1424241 | A/G | GAME-ON | 1.02 (0.98-1.07) | 0.27 | 1.04 (0.97-1.11) | 0.24 |
| rs17604349 | A/G | U4C | 1.13 (1.03-1.24) | 7.90E-03 | 1.18 (0.99-1.41) | 0.066 |
| rs17604349 | A/G | GAME-ON | 1.04 (0.99-1.08) | 0.092 | 1.02 (0.96-1.09) | 0.53 |
| rs2000999 | A/G | U4C | 0.96 (0.90-1.02) | 0.18 | 0.95 (0.84-1.08) | 0.43 |
| rs2000999 | A/G | GAME-ON | 0.99 (0.95-1.04) | 0.69 | 0.94 (0.87-1.00) | 0.054 |
| rs2072142 | T/C | U4C | 0.93 (0.88-0.97) | 1.90E-03 | 0.89 (0.82-0.97) | 6.80E-03 |
| rs2072142 | T/C | GAME-ON | 0.97 (0.94-1.01) | 0.1 | 0.95 (0.90-1.00) | 0.071 |
| rs2074626 | A/C | U4C | 0.93 (0.88-0.97) | 2.10E-03 | 0.89 (0.82-0.97) | 8.20E-03 |
| rs2074626 | A/C | GAME-ON | 0.97 (0.94-1.01) | 0.12 | 0.95 (0.90-1.01) | 0.085 |
| rs2074627 | T/C | U4C | 0.92 (0.88-0.97) | 1.60E-03 | 0.89 (0.82-0.97) | 7.30E-03 |
| rs2074627 | T/C | GAME-ON | 0.97 (0.94-1.01) | 0.12 | 0.95 (0.90-1.00) | 0.072 |
| rs236008 | T/C | U4C | 0.96 (0.90-1.03) | 0.23 | 0.93 (0.82-1.04) | 0.21 |
| rs236008 | T/C | GAME-ON | 0.98 (0.95-1.02) | 0.31 | 0.98 (0.93-1.03) | 0.49 |
| rs236009 | C/T | U4C | 0.96 (0.90-1.03) | 0.24 | 0.93 (0.82-1.04) | 0.2 |
| rs236009 | C/T | GAME-ON | 0.98 (0.95-1.02) | 0.31 | 0.98 (0.93-1.03) | 0.46 |
| rs3213422 | C/A | U4C | 1.10 (1.05-1.15) | 9.10E-05 | 1.11 (1.02-1.20) | 0.014 |
| rs3213422 | C/A | GAME-ON | 1.02 (0.99-1.06) | 0.18 | 1.07 (1.02-1.13) | 0.01 |
| rs5467 | T/C | U4C | 1.07 (0.98-1.17) | 0.13 | 1.03 (0.85-1.23) | 0.78 |
| rs5467 | T/C | GAME-ON | 1.02 (0.98-1.06) | 0.36 | 1.03 (0.97-1.10) | 0.34 |
| rs5468 | G/T | U4C | 1.04 (0.91-1.19) | 0.53 | 1.25 (0.94-1.66) | 0.13 |
| rs5468 | G/T | GAME-ON | 1.02 (0.96-1.09) | 0.47 | 1.05 (0.94-1.18) | 0.36 |
| rs6499560 | C/A | U4C | 0.92 (0.88-0.97) | 8.50E-04 | 0.89 (0.82-0.97) | 7.50E-03 |
| rs6499560 | C/A | GAME-ON | 0.97 (0.94-1.01) | 0.1 | 0.95 (0.90-1.01) | 0.093 |
| rs7195958 | G/A | U4C | 1.07 (1.01-1.12) | 0.012 | 1.08 (0.99-1.17) | 0.075 |
| rs7195958 | G/A | GAME-ON | 1.02 (0.99-1.06) | 0.18 | 1.07 (1.02-1.13) | 0.011 |
| rs8062041 | T/C | U4C | 0.91 (0.87-0.96) | 1.70E-04 | 0.89 (0.82-0.96) | 4.50E-03 |
| rs8062041 | T/C | GAME-ON | 0.97 (0.94-1.01) | 0.13 | 0.95 (0.90-1.01) | 0.089 |
| rs9941087 | A/G | U4C | 0.92 (0.88-0.96) | 2.90E-04 | 0.88 (0.82-0.96) | 2.70E-03 |
| rs9941087 | A/G | GAME-ON | 0.97 (0.94-1.01) | 0.11 | 0.95 (0.90-1.00) | 0.053 |

*The overlapping study (BPC3) was removed from the meta-analysis in the discovery phase (U4C).

OR, odds ratio; CI, confidence intervals; ER, estrogen receptor
